# Class II Malocclusion Correction Using “Distalizing Bar Appliances” and Clear Aligners: A Case Series and Clinical Technique

**DOI:** 10.3390/dj14060334

**Published:** 2026-06-01

**Authors:** Denis Bignotti, David Fracchia, Stefano Lai, Fabio Curreli, Alessio Verdecchia, Enrico Spinas

**Affiliations:** 1 Department of Surgical Sciences, Postgraduate School in Orthodontics, University of Cagliari, 09124 Cagliari, Italy; denisbignotti3@gmail.com (D.B.); david.fracchia@gmail.com (D.F.); stefano87lai@gmail.com (S.L.); f.curreli6@studenti.unica.it (F.C.); 2 Swiss Dental Clinics Group, Ardentis Clinique Dentaire, 1110 Morges, Switzerland; 3 Orthodontics Division, Instituto Asturiano de Odontología, Universidad de Oviedo, 33006 Oviedo, Spain

**Keywords:** clear aligners, Carriere, hybrid treatment, Class II

## Abstract

**Background/Objectives:** Class II malocclusion treatment in patients at the end of skeletal growth represents a significant clinical challenge. Traditional sequential distalization with clear aligners often requires prolonged treatment duration, carrying the risk of patient compliance burnout. This article describes a clinical technique combining a “Distalizing Bar Appliance” (DBA) with active lower clear aligners and Class II elastics for the management of dentoalveolar Class II malocclusion, and illustrates its application through a case series of three end-of-growth adolescent patients. **Methods:** Proposed inclusion criteria and a standardized three-phase workflow are presented: (1) distalization using a DBA supported by Class II elastics, with active lower clear aligners providing anchorage and concurrent crowding resolution; (2) alignment and arch coordination with clear aligners; and (3) finishing for occlusal settling. **Results:** In all three cases, a Class I molar and canine relationship was achieved, with cephalometric changes consistent with the dentoalveolar mechanisms previously reported for similar appliances and no clinically apparent mandibular skeletal changes. The concurrent use of active lower aligners allowed early crowding resolution, although careful monitoring of lower incisor and molar inclination was required to limit unwanted mesial tipping induced by Class II elastics. **Conclusions:** Within the limits of a case series, the technique appears clinically feasible and reproducible in carefully selected patients; comparative, controlled studies are needed before any claim of superior efficiency or effectiveness over established Class II treatment modalities can be made.

## 1. Introduction

Class II represents one of the most common malocclusions in the world population, and its treatment has always been a challenge for clinicians in orthodontic practice [1]. The treatment options for dental Class II are many, and the devices that have followed to achieve them are varied. The basis of the clinical decision is always an accurate diagnosis that identifies the causes of the malocclusion. Assessments such as the sagittal relationships of the two bone bases, the patient’s age and stage of development, the divergence and direction of growth, the relationship of the incisors to their respective bone bases and soft tissues, crowding, and the type of periodontal tissue will be necessary [2].

The most common treatment modalities, according to the severity of the Class II, described in the literature are use of intermaxillary elastics to encourage mandibular repositioning, distalizers of the upper arch, mandibular advancers such as the Herbst or Twin Block, extraoral traction, extractions, and maxillofacial surgery [2].

The “Distalizing Bar Appliance” (DBA) ranks among those appliances that produce distalization en masse of the upper arch combined with possible mandibular repositioning and mesialization of the lower elements based on patient characteristics [3]. This class of appliance was originally developed by Luis Carriere with the Carriere Motion 3D, and analogous bonded fixed Class II correctors are now commercially available from several manufacturers, for example, the Power Bar (American Orthodontics, Sheboygan, WI, USA), which share the same biomechanical concept and will hereafter be referred to collectively as “Distalizing Bar Appliances” (DBAs) [3].

**Figure 1:**
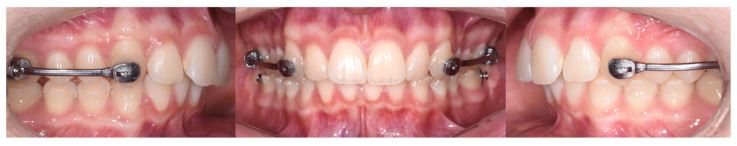
Representative intraoral photograph of a Distalizing Bar Appliance (DBA) bonded to the maxillary canine and first molar.

The DBA is a dental device (available in metal or aesthetic version) that, in its most common configuration, is designed with two bars bonded symmetrically to the upper canine and first molar. On the canine, the pad is positioned on the anterior portion of the clinical crown and is equipped with a hook attachment for securing intermaxillary elastics (Figure 1).

The first premolar is used when the canine is unerupted or malpositioned. Moving posteriorly, a specially designed pad featuring a ball-and-socket joint is bonded to the center of the first molar’s clinical crown; this configuration aids in molar rotation and distalization. To ensure stability in the lower jaw, a removable Essix-type clear retainer, modified to accommodate buccal tubes or hooks, is employed. Additional methods for anchoring the lower jaw may involve a lower lingual arch, temporary anchorage devices, or, when the patient is treated with aligners, the lower aligners themselves can be used to begin correcting the malocclusion in the lower arch concurrently with distalization [3].

The aim of this article is to describe a clinical technique combining the DBA with clear aligners for the correction of dentoalveolar Class II malocclusion, to propose inclusion criteria for appropriate patient selection, and to illustrate its application through a case series of three end-of-growth adolescent patients.

## 2. Case Presentation and Inclusion Criteria

### Inclusion Criteria

A total of three consecutive adolescent patients treated by the same operator were included in this case series. In all three cases, the distalizing appliance employed was the PowerBar Distalizing Bar Appliance (American Orthodontics, Sheboygan, WI, USA), and the clear aligners used for the lower arch during distalization, as well as for the upper and lower arches during the alignment and finishing phases, were Spark Aligners (Ormco, Brea, CA, USA). No in-office aligners were used. Patient selection was driven by the specific biomechanical requirements of the hybrid protocol. To ensure treatment predictability and safety, inclusion criteria focused on patients with a dentoalveolar Class II malocclusion supported by a normodivergent or hypodivergent skeletal pattern. The skeletal sagittal threshold adopted for inclusion was an ANB angle greater than 4°, indicating a Class II skeletal pattern of dentoalveolar relevance; the Wits appraisal and the Downs analysis were additionally evaluated to confirm the sagittal discrepancy and to exclude patients with a predominantly skeletal vertical component. This vertical control is critical to counteract the potential extrusive effects of Class II mechanics. Furthermore, the periodontal health and inclination of the lower incisors were carefully evaluated to ensure they could withstand the anchorage demands of the aligners and elastics. A detailed list of the diagnostic parameters is provided in Table 1; these criteria are consistent with the indications previously proposed for the Carriere Motion 3D and analogous fixed Class II correctors [4,5,6].

## 3. Treatment Protocol

The hybrid protocol is structured into three distinct phases to maximize efficiency and predictability.

### 3.1. Distalization Phase

This phase utilizes the “Distalizing Bar Appliance” (DBA) bonded from the maxillary first molar to the canine (or first premolar) combined with Class II elastics and active lower aligners. In this case series, the Distalizing Bar Appliance used was the PowerBar (American Orthodontics, Sheboygan, WI, USA). Throughout the text, the device will be referred to generically as the DBA.

- Digital planning: Digital models are used to select the correct appliance size. For the aligner setup, we recommend instructing the technician to “simulate a one-step distalization of the upper arch and produce only the lower aligners”. This allows for immediate lower arch coordination.
- Elastic protocol: Patients wear 6 oz ¼” elastics full-time for the first month, then increase to 8 oz 3/16” full-time from the second month. The simultaneous reduction in elastic diameter and increase in force magnitude is intended to deliver a higher initial force while maintaining a working range appropriate for the increased interarch distance that develops as distalization progresses. Patients are instructed to replace the elastics at least twice daily to maintain consistent force delivery.
- Lower anchorage and mechanics: To manage lower anchorage and resolve crowding, active lower aligners are changed every 10 days. This 10-day cadence is shorter than the standard 14-day protocol typically recommended for clear aligners and is adopted empirically with the aim of delivering the planned tooth movements within a relatively narrow time window, so that the lower arch can keep pace with the upper distalization achieved by the DBA; the choice should not be interpreted as evidence of superior anchorage, and warrants confirmation in future controlled studies. Horizontal gingival beveled attachments (3 mm) are placed from the second molar to the canine. Buttons for elastics are bonded directly to the lower first molars via aligner cut-outs.
  ○ Clinical tip: To counteract mesial tipping of the lower first molar, the button should be bonded on the mesial half of the buccal surface. Additionally, a distal tipping movement should be incorporated into the digital plan for this tooth.
- Endpoint: The phase concludes when an overcorrected Class I to a mild Class III relationship is achieved. Overcorrection is mandatory to compensate for the tipping component of the distalization and prevent relapse during the subsequent phase.
- Re-scan: We strongly advise removing the DBA before taking the intermediate scan to ensure a precise fit for the upper aligners in Phase 2.

### 3.2. Alignment Phase

During the interim period while awaiting the delivery of new aligners, patients are instructed to wear upper and lower Essix retainers featuring elastic cuts, continuing the use of 6 oz, 1/4-inch elastics exclusively at night. Once the active alignment phase commences, the primary biomechanical focus shifts to resolving any residual crowding and ensuring proper arch coordination. Should a minor Class II asymmetry persist, mild sequential distalization can be integrated into the treatment plan. Careful attention must be paid to the digital setup specifications to achieve these clinical objectives. Specifically, attachments must consistently be placed on the teeth supporting the elastics and on the maxillary first molars to maximize anchorage. To counteract the inherent retroclination effect associated with Class II mechanics, 1° of palatal root torque per aligner should be applied during retraction, ensuring the digital plan finishes with an increased positive incisor inclination. Furthermore, the setup must incorporate a digital reverse curve of Spee to finalize mandibular arch leveling and plan for a final overjet of approximately 1.5 mm to properly accommodate the thickness of the aligner material. Finally, to maintain the achieved sagittal correction throughout this active alignment period, patients are required to wear Class II elastics (6 oz, 1/4-inch) nightly, alongside an additional two hours of daytime wear.

### 3.3. Finishing Phase

The final phase of treatment is dedicated to esthetic refinement, midline coordination, and occlusal settling. To achieve these clinical objectives, light asymmetric elastics and targeted interproximal reduction (IPR) may be utilized as necessary. Upon the successful completion of active therapy, a comprehensive retention protocol is implemented. This involves the bonding of a fixed lingual retainer in the mandibular arch, extending from canine to canine (teeth 33–43), whereas the application of a maxillary fixed retainer is considered optional based on the initial clinical presentation. To ensure long-term stability, patients are also prescribed removable retainers; these are strictly for night-time wear, supplemented by an additional half-day of wear during the initial six months of the retention period.

### 3.4. Cases Presentations

The three cases presented will clinically illustrate the protocol proposed in this article. For clarity and simplicity, the diagnosis and treatment of every case are summarized in tables.

**Figure 2:**
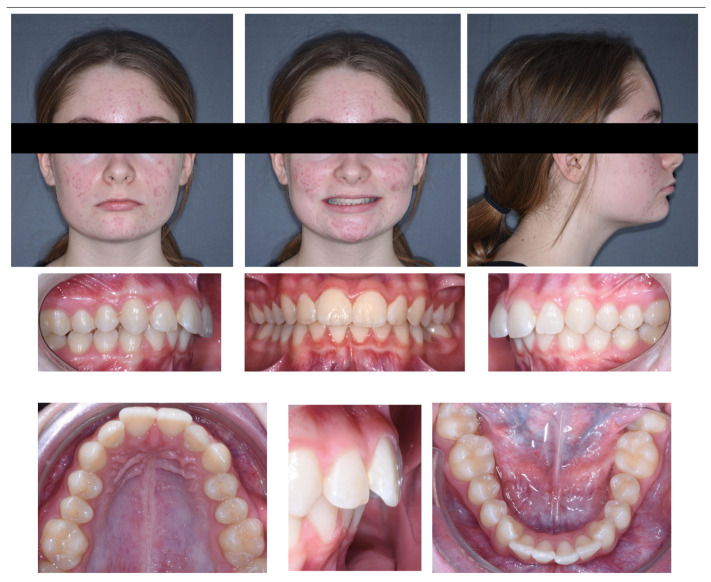
Case 1: Initial intraoral and extraoral photos.

**Figure 3:**
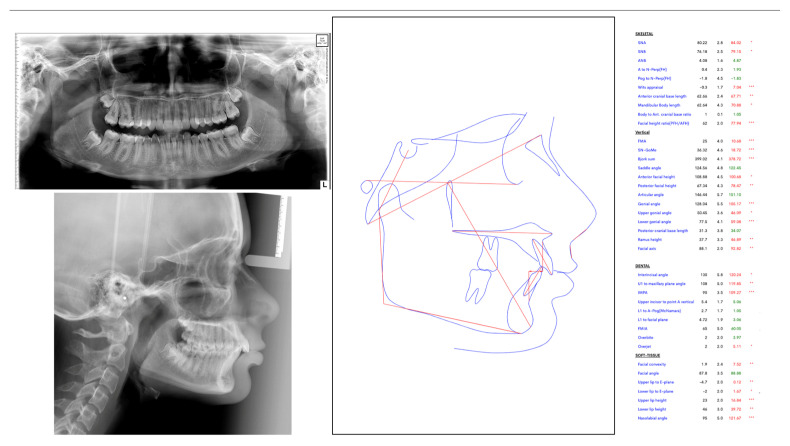
Case 1: Initial X-rays and cephalometric analysis. * = mild deviation or first level of severity from the normal value; ** = moderate deviation from the normal value; *** = marked deviation from the normal value. The greater the number of asterisks, the further the patient’s value is from the reference mean, indicating a more pronounced skeletal or dental discrepancy. These indicators are intended to help clinicians rapidly identify measurements that require greater clinical attention because they fall outside the normal range.

**Figure 4:**
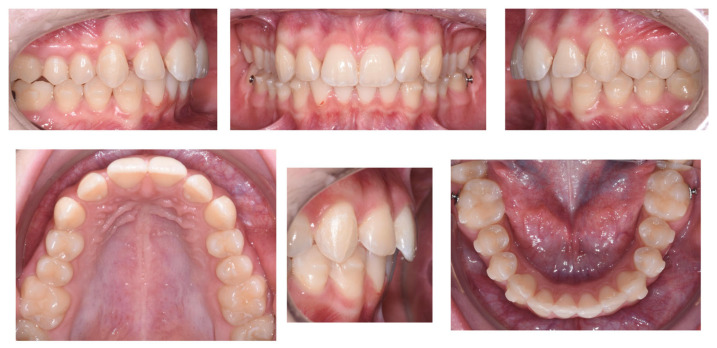
Case 1: Treatment progress, end of the distalization phase.

**Figure 5:**
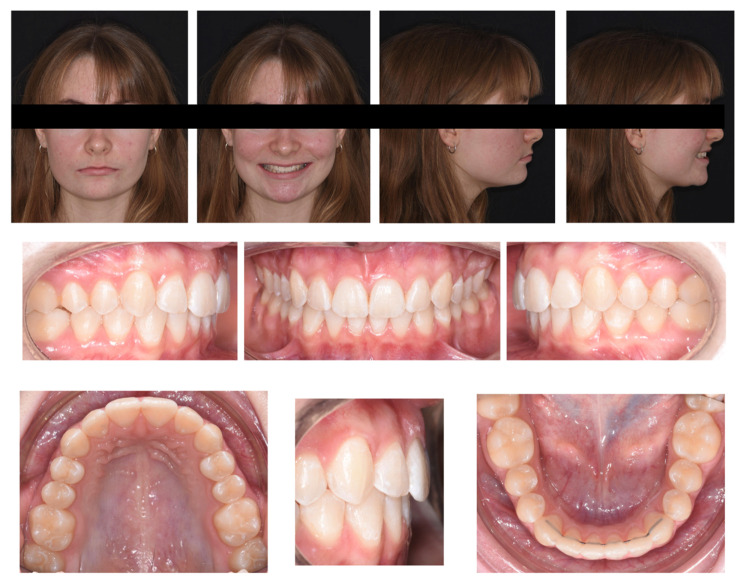
Case 1: End of the treatment, intraoral and extraoral photos.

**Figure 6:**
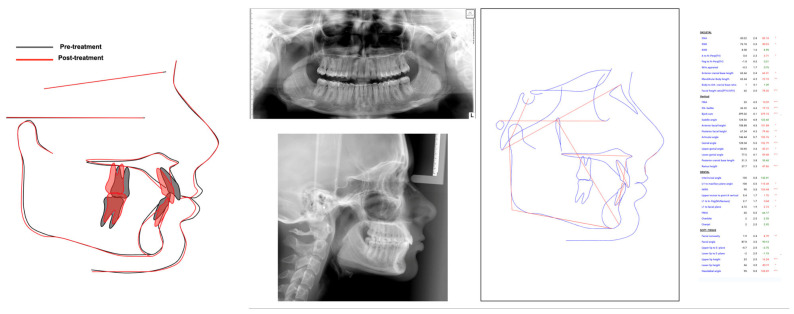
Case 1: End of the treatment, final X-rays, cephalometric analysis and super-imposition. * = mild deviation or first level of severity from the normal value; ** = moderate deviation from the normal value; *** = marked deviation from the normal value. The greater the number of asterisks, the further the patient’s value is from the reference mean, indicating a more pronounced skeletal or dental discrepancy. These indicators are intended to help clinicians rapidly identify measurements that require greater clinical attention because they fall outside the normal range.

**Figure 7:**
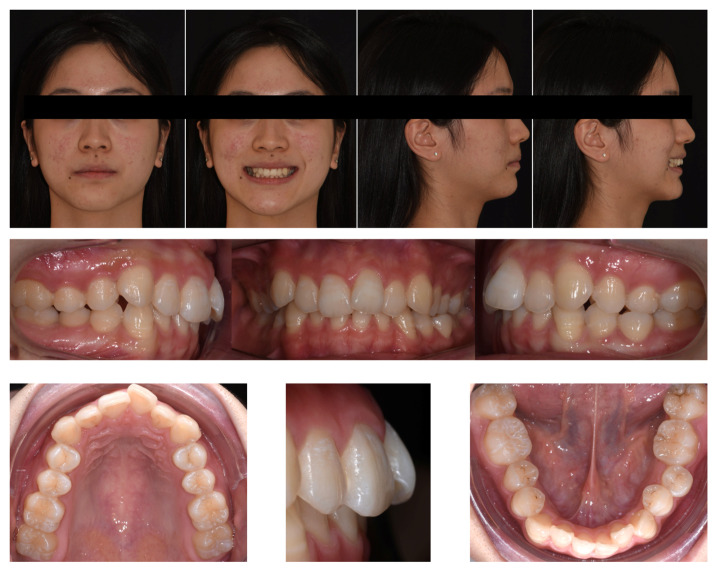
Case 2: Initial intraoral and extraoral photos.

**Figure 8:**
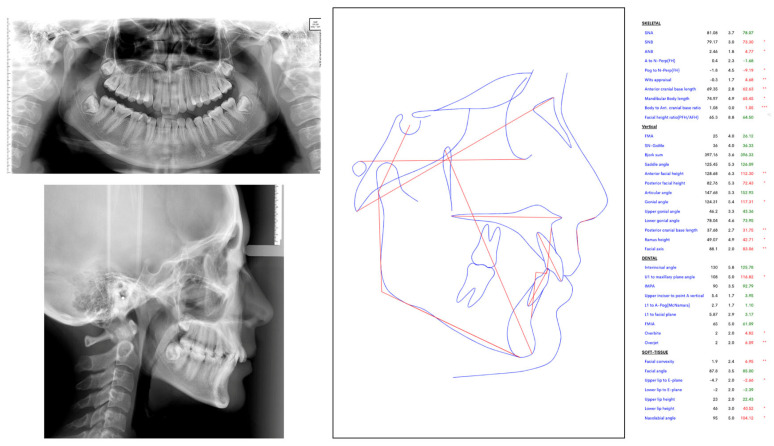
Case 2: Initial X-rays and cephalometric analysis. * = mild deviation or first level of severity from the normal value; ** = moderate deviation from the normal value; *** = marked deviation from the normal value. The greater the number of asterisks, the further the patient’s value is from the reference mean, indicating a more pronounced skeletal or dental discrepancy. These indicators are intended to help clinicians rapidly identify measurements that require greater clinical attention because they fall outside the normal range.

**Figure 9:**
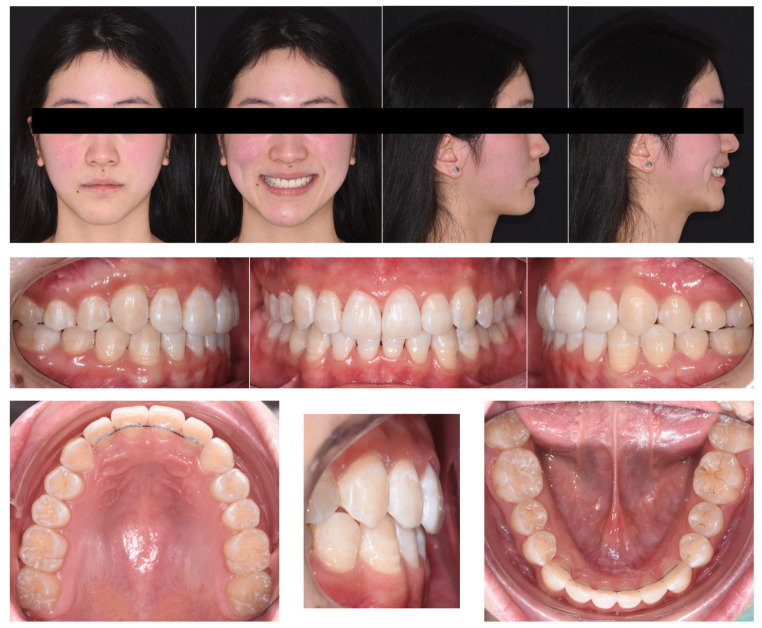
Case 2: End of the treatment, intraoral and extraoral photos.

**Figure 10:**
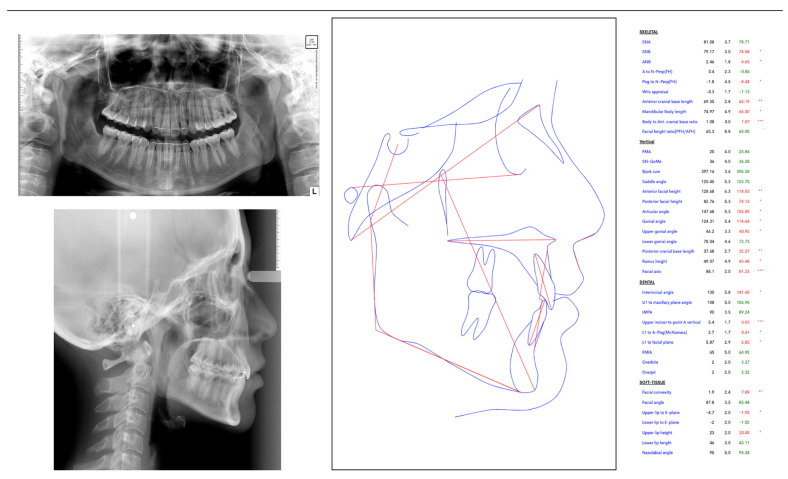
Case 2: Final X-rays and cephalometric analysis. * = mild deviation or first level of severity from the normal value; ** = moderate deviation from the normal value; *** = marked deviation from the normal value. The greater the number of asterisks, the further the patient’s value is from the reference mean, indicating a more pronounced skeletal or dental discrepancy. These indicators are intended to help clinicians rapidly identify measurements that require greater clinical attention because they fall outside the normal range.

**Figure 11:**
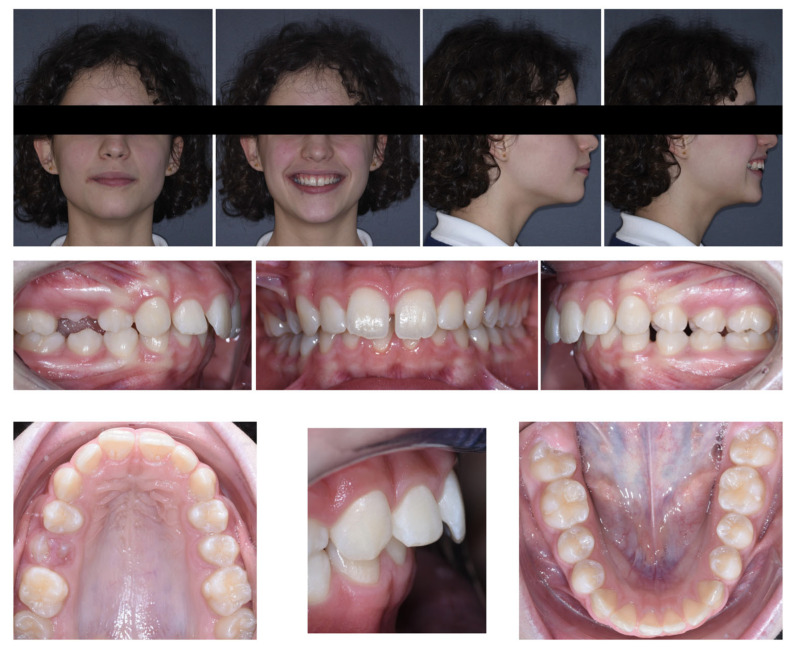
Case 3: Start of the treatment, intraoral and extraoral photos.

**Figure 12:**
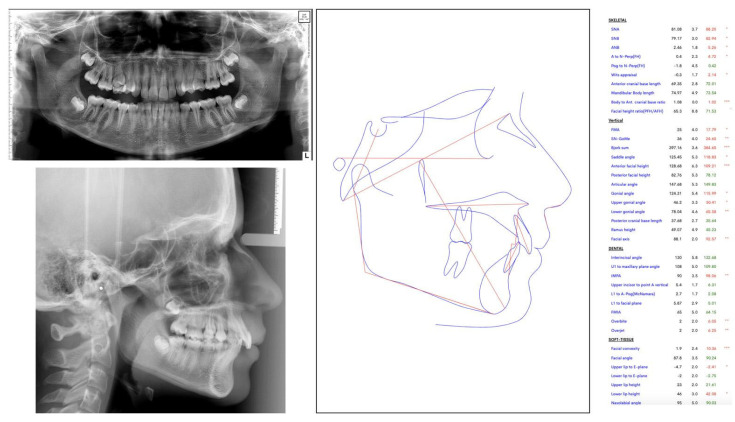
Case 3: Initial X-rays and cephalometric analysis. * = mild deviation or first level of severity from the normal value; ** = moderate deviation from the normal value; *** = marked deviation from the normal value. The greater the number of asterisks, the further the patient’s value is from the reference mean, indicating a more pronounced skeletal or dental discrepancy. These indicators are intended to help clinicians rapidly identify measurements that require greater clinical attention because they fall outside the normal range.

**Figure 13:**
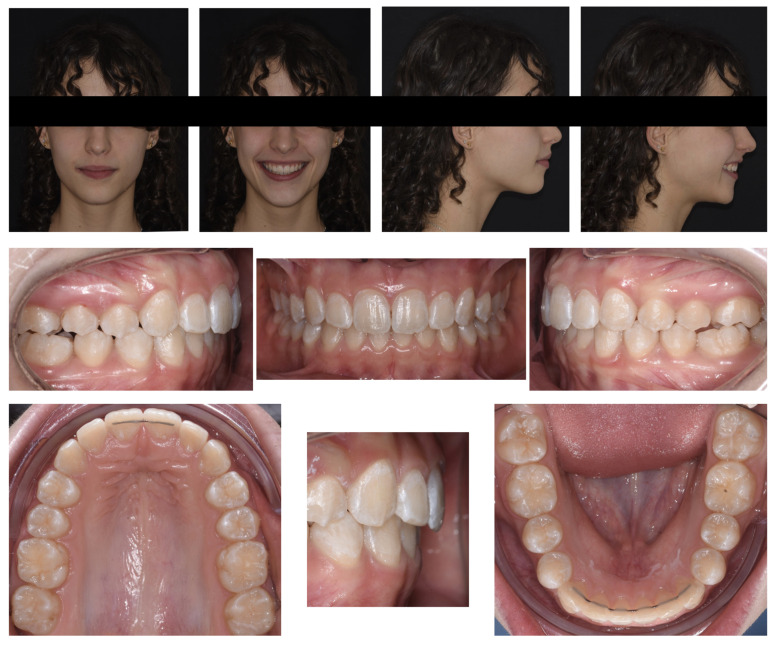
Case 3: Final intraoral and extraoral photos.

**Figure 14:**
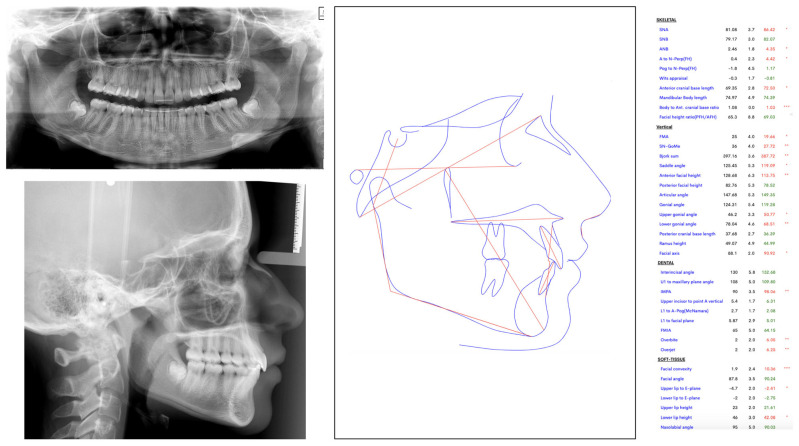
Case 3: Final X-rays and cephalometric analysis. * = mild deviation or first level of severity from the normal value; ** = moderate deviation from the normal value; *** = marked deviation from the normal value. The greater the number of asterisks, the further the patient’s value is from the reference mean, indicating a more pronounced skeletal or dental discrepancy. These indicators are intended to help clinicians rapidly identify measurements that require greater clinical attention because they fall outside the normal range.

(1) Case 1: A 15-year-old female patient presented with a Class II division 1 malocclusion, a deep bite, and a request to correct her increased overjet. Diagnosis revealed a full Class II molar relationship on the right side and a mild Class II on the left, with a non-concordant smile line. Following the digital plan, Phase 1 (distalization) was completed in 5 months. Phase 2 (alignment with clear aligners) lasted 6 months, utilizing asymmetric elastics (8 oz on the right, 6 oz on the left) to coordinate the midlines. Finishing (Phase 3) required 2 months. Total treatment time was 14 months. Post-treatment records show a bilateral Class I molar and canine relationship, correction of the deep bite, and significant aesthetic improvement (Table 2, Figure 2, Figure 3, Figure 4, Figure 5 and Figure 6).

(2) Case 2: Similar to the previous case, this 18-year-old patient sought treatment to resolve crowding and improve aesthetics (Table 3, Figure 7, Figure 8, Figure 9 and Figure 10). The diagnosis included a bilateral Class II relationship complicated by a crossbite at the second premolars (25–35) and a lower midline deviation. This case highlights the protocol’s efficiency: Phase 1 was completed in just 4 months. Phase 2 (alignment and crossbite correction) required 16 aligners over 4 months. After a 2-month finishing phase (Phase 3), the treatment concluded in a total of 12 months. A solid bilateral Class I was achieved, with full resolution of the crossbite and coincident midlines (Figure 7, Figure 8, Figure 9 and Figure 10).

(3) Case 3: A 14-year-old patient presented with generalized spacing and upper diastemas (Table 4, Figure 11, Figure 12, Figure 13 and Figure 14). While the final aesthetic result was successful, this case serves as a critical example regarding anchorage management. Phase 1 (distalization) lasted 5 months, followed by a 9-month Phase 2 (45 aligners). Total treatment time was 18 months. Although a Class I molar relationship and space closure were achieved, a specific adverse event occurred: the prolonged use of Class II mechanics resulted in mesial tipping of the lower first molars (36 and 46), which was not fully resolved at the end of treatment. To quantify the mandibular molar mesial tipping in this case, the pre- and post-treatment change in the angle formed on the lateral cephalogram by the mandibular plane (Go-Me) and the molar axis (a line passing through the root furcation and the groove separating the two buccal cusps) was calculated. The variation in this angle between pre- and post-treatment was 5.4°. This finding underscores the necessity of the reinforced anchorage protocols emphasized in the discussion section (Figure 11, Figure 12, Figure 13 and Figure 14).

## 4. Discussion

The combination of the “Distalizing Bar Appliance” (DBA) with clear aligners leverages the high level of patient compliance typically present at the onset of therapy to address the most complex component of the malocclusion: the Class II relationship. This strategy aims to address the sagittal discrepancy early in the treatment course [3]. In our three cases, Class II resolution was obtained in 4–5 months of Phase 1, consistent with the durations previously reported for the Carriere Motion 3D [4]. Although we cannot quantitatively compare treatment efficiency with sequential aligner distalization within a case series design, the latter is known to require prolonged aligner wear and is associated with the risk of “compliance burnout” in adolescent patients [7]. Compared with the previously described Carriere–aligner protocols [4], which employed a passive Essix-type appliance for lower-arch stabilization during distalization, the protocol presented here uses active lower clear aligners as a dual-purpose tool: simultaneously providing dentoalveolar anchorage and resolving lower-arch crowding from the outset. The systematized three-phase scheme, with defined biomechanical endpoints, attachment design, and elastic protocol, is intended to formalize the clinical workflow of this combined approach. Furthermore, the literature suggests that fixed Class II correctors like the DBA offer greater comfort and a more positive patient experience for adolescents compared to other intramaxillary anchorage devices [8].

A key advantage of this hybrid protocol is the concurrent use of lower aligners. This allows the clinician to initiate lower crowding resolution immediately while providing resistance to the mesializing force of the Class II elastics. However, anchorage management remains critical. Class II intermaxillary elastics are well known to produce undesired lower incisor proclination and mesial movement of the lower dentition, and this side effect must be actively counteracted through digital setup planning and attachment design [9]. As observed in Case 3, prolonged elastic use can lead to mesial tipping of the lower molars. To mitigate this side effect, our updated protocol incorporates specific digital features: placing the button on the mesial half of the lower molar and programming distal tipping in the digital setup.

Cephalometric superimpositions from our case series confirm that the Class II correction is primarily dentoalveolar rather than skeletal. The effects include distalization, distal tipping, and distorotation of the upper first molars, accompanied by mesialization of the lower molars and a clockwise rotation of the occlusal plane. Upper canines and premolars also exhibit distal movement with tipping and extrusion. These findings are consistent with the current literature [5,10,11]. At the end of Phase 1, all three cases had reached a Class I molar and canine relationship and no residual translational distalization needed to be programmed in the subsequent upper aligner stage; only an additional distal rotation of the upper first molars, ranging from 3° to 7°, was scheduled in the digital setup of Phase 2 to refine molar angulation and arch coordination. This indicates that, in the cases presented here, the DBA combined with Class II elastics was sufficient to deliver the full sagittal correction during Phase 1, with the upper aligners limited to rotational and alignment refinements rather than continued distalization. Notably, recent systematic evidence indicates that the predictability of rotational movements with clear aligners remains limited, particularly for round-rooted teeth, and is improved by the use of dedicated attachments, interproximal reduction, and overcorrection in the digital setup [12]. Consistent with the literature [6], our cases did not show clinically apparent mandibular skeletal changes attributable to the appliance, supporting the interpretation that the Class II correction obtained with the DBA is achieved primarily through dentoalveolar, rather than orthopedic, mechanisms.

The vertical effects of these mechanics—specifically the extrusion of upper incisors and molars—highlight the importance of careful diagnosis. The clockwise rotation of the occlusal plane aids in correcting the Class II relationship but dictates caution in hyperdivergent patients or those with an open bite tendency [6]. Specific side effects described in the literature, such as upper molar constriction and canine extrusion [6,10], can be managed by strategic bonding placement (more occlusal) and overcorrection in the digital plan.

When selecting the treatment strategy, other valid options were considered. Sequential distalization with aligners is the most common alternative but it relies heavily on prolonged aligner wear [13]. Skeletal anchorage (TADs) offers distalization without compliance but involves higher costs and invasiveness [14]. Extractions were excluded in these cases due to the patients’ facial profiles and moderate crowding [15]. Ultimately, the DBA–aligner hybrid approach was deemed the most practical choice, offering an optimal balance between biological cost, treatment time, and patient motivation.

## 5. Conclusions

Within the limits of a case series of three end-of-growth adolescent patients, the following conclusions can be drawn. First, the hybrid technique combining the Distalizing Bar Appliance (DBA) with active lower clear aligners and Class II elastics allowed correction of dentoalveolar Class II malocclusion to a Class I molar and canine relationship in all three cases, with cephalometric changes consistent with the dentoalveolar mechanisms previously reported for similar appliances, and with no clinically apparent mandibular skeletal changes attributable to the device. The proposed inclusion criteria and the systematized three-phase workflow, distalization, alignment, and finishing, appear clinically applicable and reproducible. Second, this case series cannot demonstrate that the described technique is more efficient, faster, or more effective than alternative Class II strategies such as sequential aligner distalization, fixed functional appliances, skeletal anchorage, or extraction-based mechanics: the absence of a comparison group, the small sample size, the heterogeneity of baseline malocclusions, and the lack of long-term retention data preclude any inference of superiority. Third, to test whether the apparent clinical advantages translate into measurable benefits, the technique should be evaluated in prospective controlled studies with a priori-defined inclusion criteria, standardized outcome measures (treatment duration, cephalometric and digital model superimpositions, periodontal indices, patient-reported outcomes, and stability at ≥1 year of retention), and a direct comparison with at least one established Class II treatment modality. Until such data become available, the protocol described here should be regarded as a clinical technique whose feasibility is supported by these three cases, rather than as a method of proven superiority.

## Figures and Tables

**Table 1:** Diagnostic inclusion criteria.

DIAGNOSTIC INCLUSION CRITERIA	
Aesthetic	Proportioned profile with good mandible’s projection, chin shape, normal dimension of the note. Lower lips can be slightly protruded or in good position with a normal width.
Skeletal class	Class I skeletal or moderate Class II skeletal pattern with adequate length of the body and mandibular ramus.
Skeletal divergence	Normodivergent or hypodivergent patient.
Molar dental class	At least one side in Class II, end-to-end, or with the palatal cusp of the upper sixth resting more mesially than the fossa of the lower first molar.
Canine molar class	Class II or ectopic canine.
Rotation of upper first molars	Mesiorotated.
Overjet	Increased.
Overbite	Normal or increased.
Upper incisors	Preferably proclined.
Lower incisors	With potential for proclination or in good position relative to the symphysis and the mandibular plane.
Transverse diameter of the upper arch	Good or mildly reduced. Avoid patients with a very contracted arch or in crossbite.
Upper crowding	From mild to severe.
Lower crowding	Mild or with potential for proclination.
Growth	If present, it must be favorable (normal or in anterotation).

**Table 2:** Summary of Case 1.

Section	Details
**Diagnosis and Etiology**	- **Age**: 15, **Patient’s request**: Correction of deep bite and increased overjet.
	- **Extraoral Frontal Examination**: Square face type, mandibular deviation to the left, non-concordant smile line with the lower lip, Upper incisive midline deviated to the left relative to the facial midline, Occlusal cant anti-clockwise.
	- **Lateral View**: Flat profile, retruded mandible, normal nasolabial angle, good chin and nose projection.
	- **Intraoral Examination**: full Class II on the right side, mild Class II on the left, upper midline deviation, increased overbite and overjet, accentuated Spee curve.
	- **Cephalometric Examination**: Confirmed extraoral findings, incisors well-positioned in bone bases, definitive teeth visible (including wisdom tooth germs, no space to erupt).
**Treatment Objectives**	- **Main Goals**: Correct Class II malocclusion, obtain a correct overbite and overjet, improve smile aesthetics and harmonize smile with lips.
**Treatment Protocol and Progress**	- **Digital Planning**: Entire treatment planned digitally.
	- **Phase 1**: 5 months.
	- **Phase 2**: 22 aligners for both arches, 5–6 months (8 oz elastic on the right, 6oz on the left).
	- **Phase 3**: 8 aligners, lasted 2 months.
	- **Total Treatment Time**: 14 months, including wait time for new aligners.
**Results**	- **Achieved Results**: Bilateral first class, overjet and overbite correction, midlines centered with facial midline.
	- **Aesthetic Improvement**: Smile aesthetics improved, good relationship between lips and teeth, and preserved facial profile proportions.
	- **Orthodontic Movements**: Distalization, distal tipping and distorotation of upper first molars, mesialization of lower arch, post-rotation of occlusal plane favoring class correction.
	- **Cephalometric Findings**: Distalization/distal tipping of upper first molars and mesialization of lower molars, intrusion of upper molars, extrusion of lower molars, correction of Spee curve, retroinclination and extrusion of upper incisors, occlusal plane post rotation.

**Table 3:** Summary of Case 2.

Section	Details
**Diagnosis and Etiology**	- **Age**: 18, **Patient’s request**: Correction of tooth crowding for aesthetic improvement.
	- **Extraoral Frontal Examination**: Oval face type, slight mandibular deviation to the right, non-concordant smile line with the lower lip, upper midline centered with the face, lower midline deviated to the right. Occlusal cant clockwise.
	- **Lateral View**: Flat profile, good sagittal position of maxillae, normal nasolabial angle, good chin and nose projection, good anterior limit of dentition at smile.
	- **Intraoral Examination**: Bilateral Class II head-to-head, lower midline deviation, increased overbite and overjet, upper and lower crowding, accentuated Spee curve, crossbite at 35–25.
	- **Cephalometric Examination**: Confirmed extraoral findings, incisors well-positioned in bone bases.
**Treatment Objectives**	- **Main Goals**: Correct Class II malocclusion, resolve crowding, good overbite and overjet, resolve crossbite at 25–35, improve smile aesthetics, and harmonize smile with lips.
**Treatment Protocol and Progress**	- **Digital Planning**: Entire treatment planned digitally.
	- **Phase 1**: 4 months.
	- **Phase 2**: 16 aligners for both arches, 4 months.
	- **Phase 3**: 8 aligners, lasted 2 months.
	- **Total Treatment Time**: 12 months, including wait time for new aligners.
**Results**	- **Achieved Results**: Bilateral first class, overjet and overbite correction, inferior and superior crowding resolved, crossbite at 25–35 corrected, midlines centered with facial midline, partial occlusal cant reduction.
	- **Aesthetic Improvement**: Smile aesthetics improved, good relationship between lips and teeth, and preserved facial profile proportions.
	- **Orthodontic Movements**: Distalization, distal tipping and distorotation of upper first molars, mesialization of lower arch, post-rotation of occlusal plane favoring class correction.
	- **Cephalometric Findings**: Distalization/distal tipping of upper first molars and mesialization of lower molars, intrusion of upper molars, extrusion of lower molars, correction of Spee curve, retroinclination and extrusion of upper incisors.

**Table 4:** Summary of Case 3.

Section	Details
**Diagnosis and Etiology**	- **Age**: 14, **Patient’s request**: Closing the upper diastemas.
	- **Extraoral Frontal Examination**: Oval face type, slight mandibular deviation to the right, non-concordant smile line with the lower lip, upper midline centered with the face.
	- **Lateral View**: Good profile, good sagittal position of maxillae, normal nasolabial angle, good chin and nose projection, good anterior limit of dentition at smile.
	- **Intraoral Examination**: Bilateral Class II head-to-head, increased overbite and overjet, upper and lower spacing, accentuated Spee curve.
**Treatment Objectives**	- **Main Goals**: Correct Class II malocclusion, resolve crowding, good overbite and overjet, improve smile aesthetics, close the diastemas.
**Treatment Protocol and Progress**	- **Digital Planning**: Entire treatment planned digitally.
	- **Phase 1**: 5 months.
	- **Phase 2**: 45 aligners on both arches, 9 months.
	- **Phase 3**: 12 aligners, lasted 3 months.
	- **Total Treatment Time**: 18 months, including wait time for new aligners.
**Results**	- **Achieved Results**: Bilateral first class, overjet and overbite correction, inferior and superior spacing resolved. Collateral dental effect for the use of Class II elastics: mesial tipping of 36 and 46 that has not completely been resolved.
	- **Aesthetic Improvement**: Smile aesthetics improved, good relationship between lips and teeth, and preserved facial profile proportions.
	- **Orthodontic Movements**: Distalization of upper first molars, mesialization of lower arch, post-rotation of occlusal plane favoring class correction.
	- **Cephalometric Findings**: mesialization of lower molars, correction of Spee curve, retroinclination of upper incisors.

## Data Availability

Original contributions presented in the study are included in the article. Further inquiries can be directed to the corresponding authors.
