# Correction: Bignotti et al. Class II Malocclusion Correction Using “Distalizing Bar Appliances” and Clear Aligners: A Case Series and Clinical Technique. *Dent. J.* 2026, *14*, 334

**DOI:** 10.3390/dj14060372

**Published:** 2026-06-16

**Authors:** Denis Bignotti, David Fracchia, Stefano Lai, Fabio Curreli, Alessio Verdecchia, Enrico Spinas

**Affiliations:** 1 Department of Surgical Sciences, Postgraduate School in Orthodontics, University of Cagliari, 09124 Cagliari, Italy; 2 Swiss Dental Clinics Group, Ardentis Clinique Dentaire, 1110 Morges, Switzerland; 3 Orthodontics Division, Instituto Asturiano de Odontología, Universidad de Oviedo, 33006 Oviedo, Spain

The authors would like to include an acknowledgment in the original publication [1].

A correction has been made by adding the following Acknowledgments section:

**Acknowledgments:** We express our deepest gratitude to the Centro Ortodontico Vicentino and its director, Leonella Caliari, for their invaluable collaboration, clinical expertise, and essential support throughout this study.

The authors state that the scientific conclusions are unaffected. This correction was approved by the Academic Editor. The original publication has also been updated.
